# Illuminating the Disease Landscape: Human Oxidoreductase-Targeted Imaging and Quantification Agents for Precision Imaging and Activity Determination

**DOI:** 10.1021/acs.accounts.6c00462

**Published:** 2026-09-23

**Authors:** Nasim Akhtar, Stephy Jomy, Shibalik Mukherjee, Robin L. McCarley

**Affiliations:** † The Fralin Life Sciences Institute, Virginia Tech, Blacksburg, Virginia 24061, United States of America; ‡ Department of Chemistry, Virginia Tech, Blacksburg, Virginia 24061, United States of America; ∥ Sterling Pharma Solutions, Germantown, Wisconsin 53022, United States of America

## Abstract

Electron-specific reduction reactions afforded by quinone oxidoreductases have long been recognized as essential for safeguarding *healthy* mammalian cells against oxidative stress induced by environmental insults. The presence of reactive oxygen species (ROS) initiates the upregulation of intracellular redox enzyme activities used to battle the effects of ROS in healthy cells. At the same time, *diseased* human cells hijack the pathways of certain oxidoreductases, such as the two-electron-specific human NAD­(P)­H:quinone oxidoreductase isozyme 1 (hNQO1), and use the overexpressed enzymes as standalone weapons or in concert with other proteins as an arsenal to advance disease progression and defeat therapies. Fortunately, in numerous cancers, inherent overexpression of hNQO1 activity is successfully targeted to trigger quinone-bearing, pro-therapeutic agents whose hNQO1 reduction yields cytotoxic agents only within hNQO1 activity-overexpressing cells. Furthermore, the development of diverse new approaches for controlling and leveraging expression of intracellular hNQO1 activity for disease treatment is being propelled by the growing evidence about the significant role of hNQO1 in cardiovascular diseases, central nervous system disorders, and autoimmune conditions.

While oxidoreductases like hNQO1 are pivotal in many diseases and their development, there is a limited toolset for nonintrusive, selective, and low-limit-of-detection determination and quantification of enzyme activity in human tissue or mammalian models of disease. Our research team used tailored design criteria and computational routes to develop fluorescence-based molecular probes for highly selective, rapid measurement and visualization of hNQO1 activity through the latter’s catalyzed production of reporters that are spectrally distinct from their parent probes. In this Account, we report on low- and tunable-brightness probes designed to be straightforwardly made and offer rapid intracellular accessibility to and fast activation by target enzymes with high selectivity to generate high-brightness reporters having large Stokes shifts. The probe/reporter pairs exhibit high photophysical, chemical, and environmental stabilities, low cellular toxicities, and emission energies that enable mucosal/epithelial and subsurface tissue imaging. These off–on and on–on probe–reporter pairs afford determination of intracellular enzyme presence and activity in simple cell cultures, complex tumor mimics, and an animal model of human cancer. These approaches offer unparalleled image contrast (target-to-background ratio, TBR), dynamic range, and limit of detection. The pivotal results presented in this Account stem from the focused development of fluorogenic molecular probes designed to assess hNQO1 activity across diverse cellular structures. The generalizable strategies for probe development described herein have broad implications for chemical biology and diagnostic/theranostic imaging with significant translational potential spanning various diseases.

## Graphical abstract

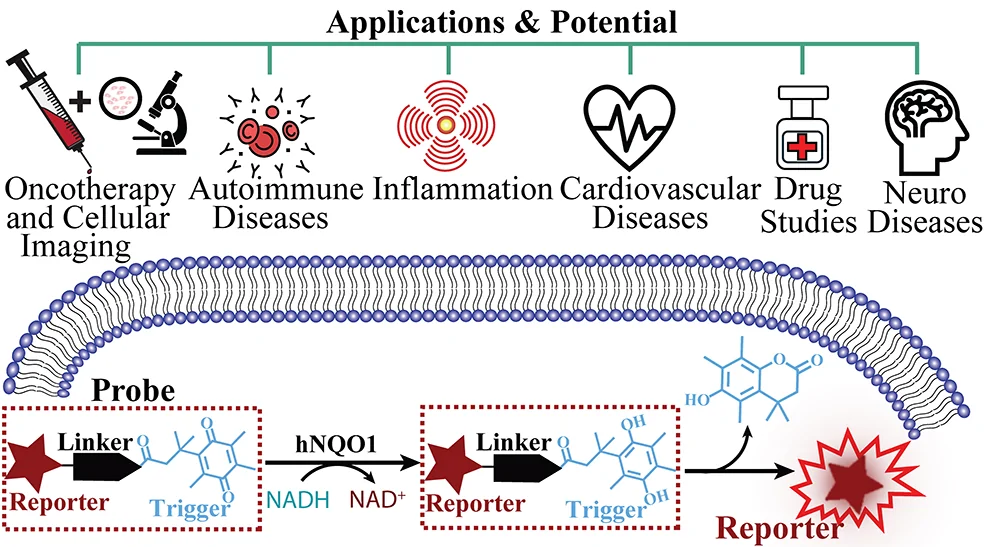

## Key References

-

Silvers, W. C. ; Prasai, B. ; Burk, D. H. ; Brown, M. L. ; McCarley, R. L. Profluorogenic Reductase Substrate for Rapid, Selective, and Sensitive Visualization and Detection of Cancer Cells That Overexpress NQO1. J. Am. Chem. Soc. 2013, 135 (1), 309–314; DOI: 10.1021/ja309346f.PMC470311223198810 ^1^ Described here are the design and evaluation of a newly synthesized turn-on probe whose intense fluorescent reporter signature is revealed only through highly selective probe activation by intracellular hNQO1 present in tumor cells of multiple origins.
-

Hettiarachchi, S. U. ; Prasai, B. ; McCarley, R. L. Detection and Cellular Imaging of Human Cancer Enzyme Using a Turn-On, Wavelength-Shiftable, Self-Immolative Profluorophore. J. Am. Chem. Soc. 2014, 136 (21), 7575–7578; DOI: 10.1021/ja5030707.PMC404675424813575 ^2^ This work concerns the first fluorogenic reporter and probe pair with distinct optical properties resulting from an electronic cascade linker between the hNQO1 substrate and the reporter, giving rise to groundbreaking measurement figures of merit upon hNQO1 activation of probe.
-

Best, Q. A. ; Johnson, A. E. ; Prasai, B. ; Rouillere, A. ; McCarley, R. L. Environmentally Robust Rhodamine Reporters for Probe-Based Cellular Detection of the Cancer-Linked Oxidoreductase hNQO1. ACS Chem. Biol. 2016, 11 (1), 231–240: DOI: 10.1021/acschembio.5b00792.26555574 ^3^ Strategic design and synthesis yielded low-energy-emissive rhodamine probe/reporter pairs that are not altered by intracellular NADH levels, while being rapidly and specifically activated by target hNQO1 vs other oxidoreductases.
-

Nakamura, Y. ; Shen, Z. ; Harada, T. ; Nagaya, T. ; Sato, K. ; Okuyama, S. ; Ogata, F. ; Choyke, P. L. ; McCarley, R. L. ; Kobayashi, H. Characteristics of Ovarian Cancer Detection by a Near-Infrared Fluorescent Probe Activated by Human NAD­(P)­H:Quinone Oxidoreductase Isozyme 1 (hNQO1). Oncotarget 2017, 8, 61181–61192; DOI: 10.18632/oncotarget.18044.PMC561741528977855 ^4^ Using a preclinical animal model of human ovarian cancer, it is shown that a low-energy-light-emissive probe/reporter pair allows for identification and surgical removal of peritoneally disseminated micrometastases with the figures of merit pointing to potential for identification of cancerous lesions deep within tissue.

## 1 Introduction

Identifying protein expression is only a starting point; functional insight mandates the measurement of activity. In complex biological systems, particularly diseased tissues, enzyme abundance often fails to reflect catalytic function linked to cofactors, inhibitors, redox balance, compartmentalization, and microenvironmental effects. This is especially true for redox enzymes, whose roles shift with context, supporting antioxidant pathways in healthy cells, while contributing to pathology under stress. Motivated by the challenge of addressing the great need to determine enzyme activity in living systems,^5,6^ we developed chemical probes that report enzyme activity in situ and in real time with minimal perturbation.

Human NAD­(P)­H:quinone oxidoreductase isozyme 1 (hNQO1) is a compelling target for activity-based sensing because of its biologically central and chemically exploitable properties.^7–10^ Its catalyzed two-electron reduction of quinones to hydroquinones avoids radical-generating redox cycling and contributes to oxidative stress defense and redox homeostasis.^11,12^ Also, hNQO1 is markedly overexpressed in diverse tumors, including colon, lung, pancreas, breast, liver, stomach, kidney, and ovarian cancers.^1,11,13–17^ Furthermore, hNQO1 regulates protein stability through controlling 20S proteasomal degradation pathways involving tumor suppressors such as p53, p73α, and p33,^18^ while stress-response pathways, including Nrf2, further modulate its activity.^19^ Together, these features position hNQO1 as key therapeutic and biomarker targets with its redox chemistry auguring well for transduction of enzymatic activity into optical signals.

Our design considerations regarding activation and selectivity are summarized schematically in Figure 1, which illustrates hNQO1-responsive probe activation in hNQO1-positive and -negative cells, together with broader applications in disease detection, activity imaging, pathway mapping, drug-response studies, and fluorescence-guided surgery (FGS). These studies demonstrate how enzymatic activity can be converted to quantifiable optical signals in living systems.

**Figure 1:**
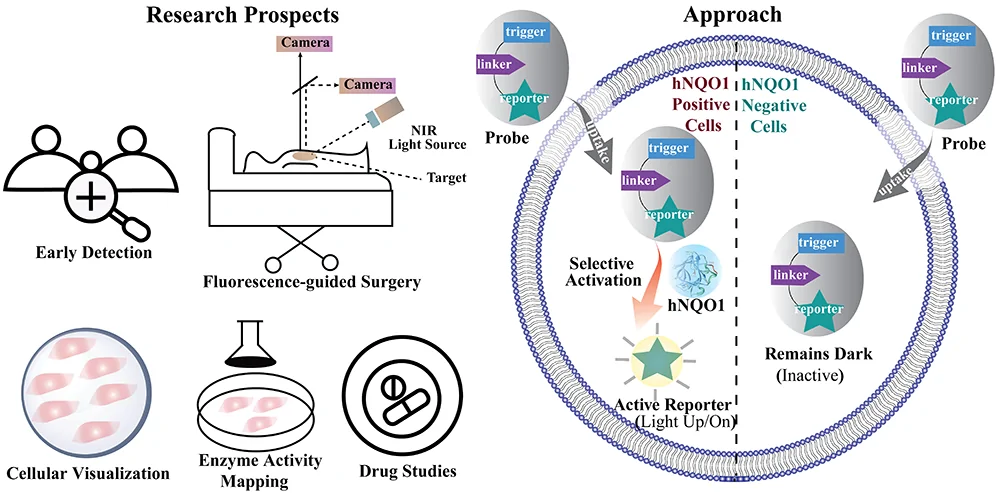
Biomedical goals and conceptual activation strategy for hNQO1-activatable fluorogenic probes.

Designing effective activatable probes requires balancing activation efficiency and selectivity in the face of required photochemical traits. Probes must efficiently enter cells, access cytosolic hNQO1, and activate rapidly, while also remaining stable to minimize nonspecific responses. This requires approaches that integrate enzymatic reactivity, chemical stability, solubility, intracellular accessibility, selectivity against competing reductases, and reporter photophysics. This Account traces the evolution of these strategies from foundational trigger–linker architectures^1^ to wavelength tuning,^2^ improved contrast, and validation in increasingly realistic models, including three-dimensional tumor mimics and preclinical models.^4,20^

The trajectory of this research program is summarized in Figure 2, wherein are highlighted probe architectures developed by the McCarley group and improved generations with refined relationships between enzymatic activation, photophysical output, and biological performance.^1–4,20–22^ Together, these studies demonstrate how integrating enzymology, synthetic chemistry, and fluorescence spectroscopy enables sensitive and selective activity-based imaging in complex biological environments.

**Figure 2:**
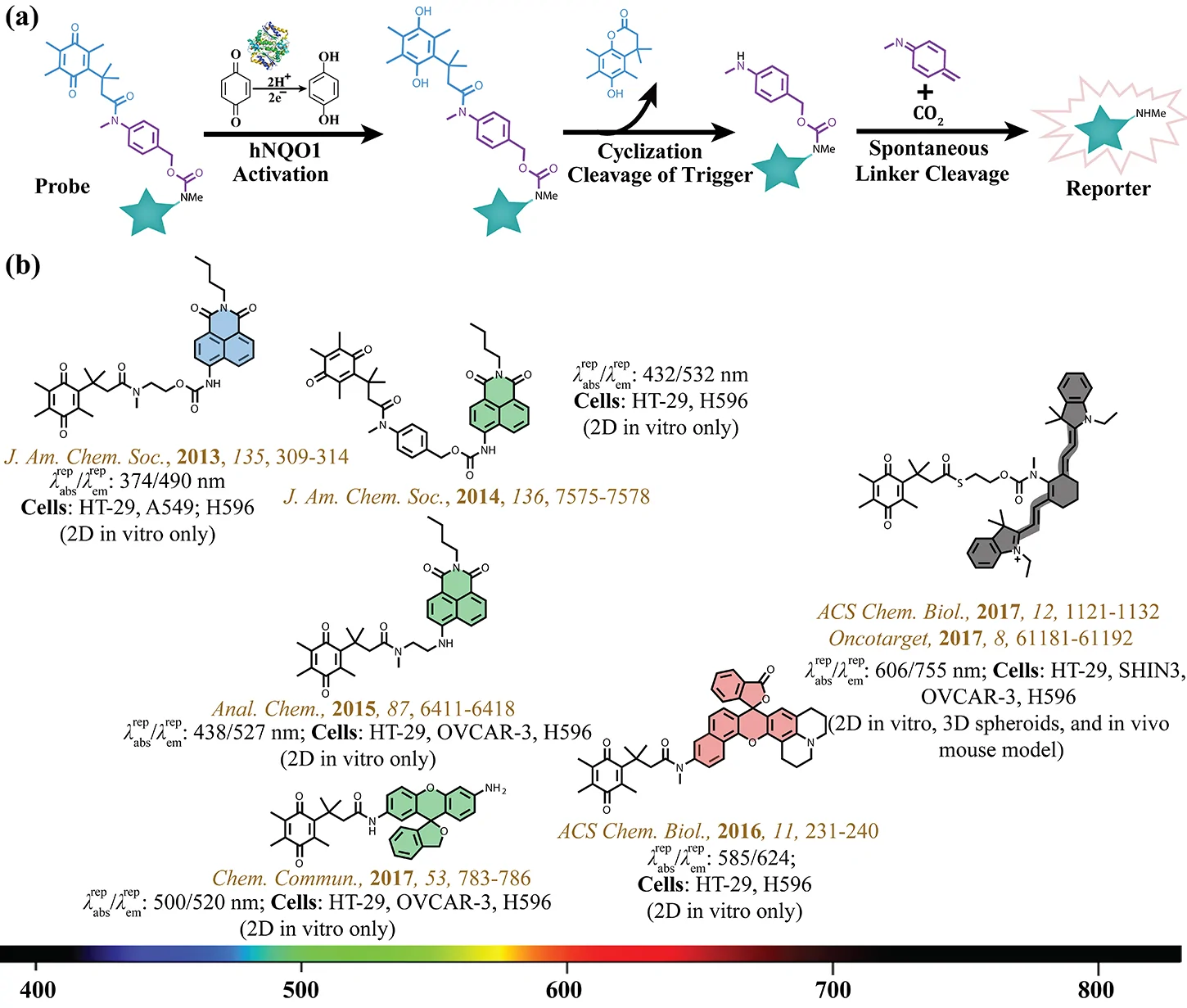
Evolution of hNQO1-responsive fluorogenic probes developed by the McCarley group. (a) General activation strategy for hNQO1-responsive probes. (b) Probes developed in the McCarley group illustrate the evolution of hNQO1-activatable fluorogenic probes. Maximum wavelength of reporter absorption (λ_abs_^rep^) and emission (λ_em_^rep^) are provided.

While the initial development of hNQO1-responsive probes was driven by tumor imaging, their potential extends to inflammatory diseases,^23^ cardiovascular injury,^24^ metabolic disorders,^25^ and neurodegenerative conditions,^26–28^ where oxidative stress is prominent. Thus, hNQO1 serves not only as a cancer biomarker but also as a window into the dynamic regulation of cellular redox biology. A central goal of this Account is to define design principles for enzyme-activatable probes capable of resolving biochemical activities in living systems.

With this foundation, the next question becomes practical: What features must hNQO1-responsive probes possess to function effectively in complex biological environments?

## 2 Design Requirements for an Ideal hNQO1 Probe/Reporter Pair

It is crucial to define the key performance criteria of enzyme-responsive probes that are effective in biological environments. While enzyme-triggered activation is fundamental, practical imaging performance depends on physicochemical, biological, and photophysical properties that determine whether a probe can deliver reliable, high-contrast readouts.

An effective probe/reporter pair must balance chemical and photophysical stability with high imaging contrast (i.e., a high target-to-background ratio, TBR). This requires efficient cellular uptake, low cytotoxicity, and rapid, selective response to the target enzyme. From an optical standpoint, high TBR is partly achieved by high reporter brightness (ε_rep_Φ_rep_) and minimal probe background (ε_probe_Φ_probe_). A large Stokes shift augments detection via reduced spectral overlap and scatter. For in vivo applications, probes must exhibit chemical and environmental stability and minimal interference with endogenous biological processes, Figure 3.

**Figure 3:**
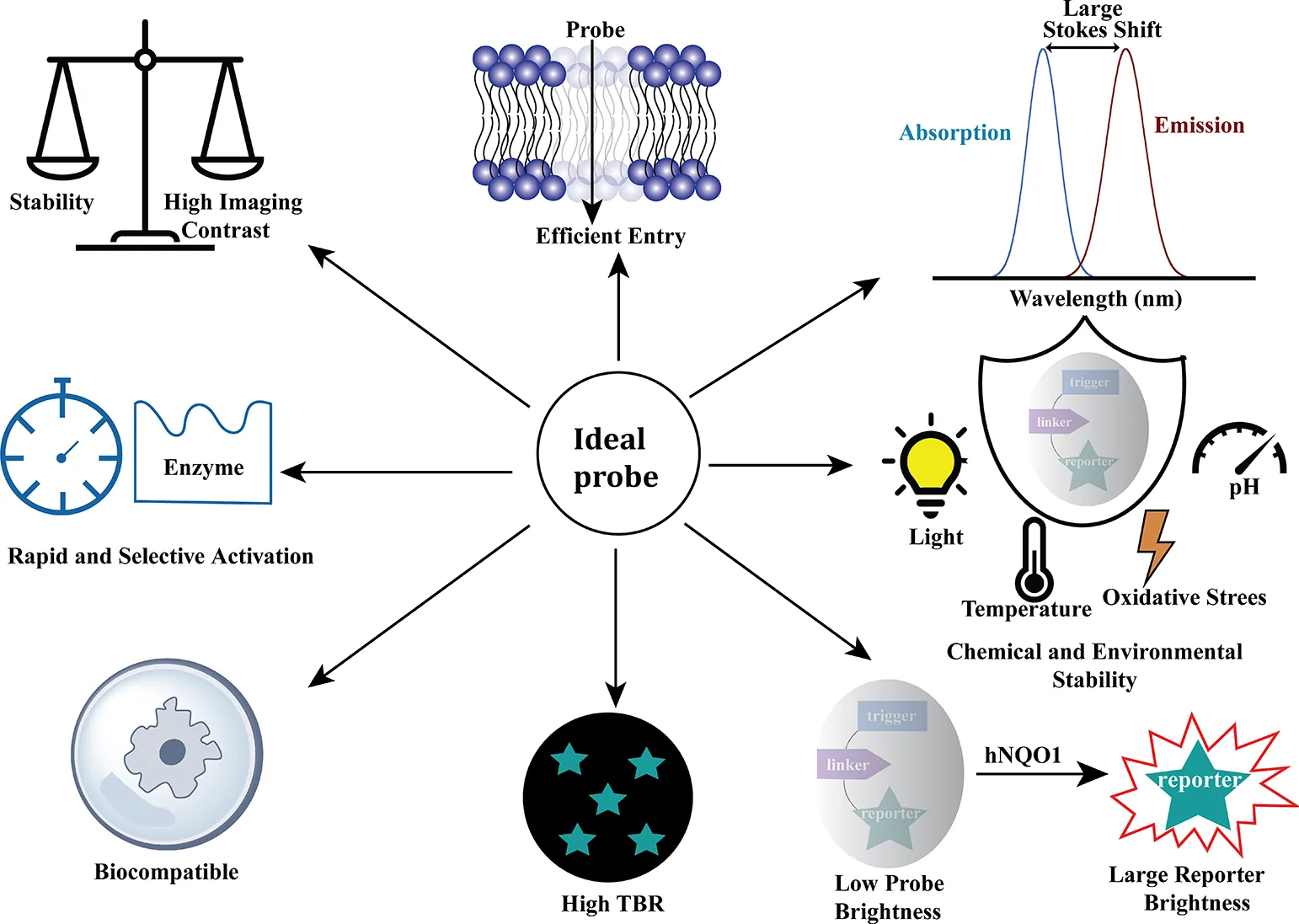
Conceptual overview of the essential attributes of an ideal activatable probe, highlighting performance, stability, selectivity, and translational considerations.

Among these design elements, the enzyme-responsive trigger is particularly critical as it governs both activation efficiency and selectivity. In hNQO1-based systems, this has led to the widespread use of quinone-based triggers, particularly the **Q**_**3**_ trimethyl lock platform, which plays a central role in probe design.

## 3 Mechanistic Basis of Quinone Triggering and Trimethyl-Lock Release

Once the design requirements of hNQO1-responsive probes are established, the next question is, which chemical architectures best satisfy them? Quinone-based triggers (**Q**_**3**_) are particularly effective because they exploit the two-electron reduction chemistry of hNQO1 while enabling efficient downstream reporter generation.^29^

The upregulation of hNQO1—together with its roles in radical scavenging,^30^ stabilization of tumor suppressor proteins,^31^ and quinone detoxification^32^—has established it as a popular biomarker for targeted antitumor strategies.^33^ Leveraging hNQO1-catalyzed quinone reduction, numerous activatable probes incorporating quinone triggers have been developed^1–4,21,29^ with the **Q**_**3**_ trimethyl-lock platform offering distinctive advantages.^34^ Methyl substitution restricts conformational flexibility of the hydrocoumarinic acid scaffold, facilitating access to the transition state,^35^ and underpins relief of steric strain during lactonization (Thorpe–Ingold effect), which accelerates reaction rates by up to ∼10^7^ compared to unsubstituted analogs.^36^ This hydrocoumarinic system serves as a model for how structure enhances trigger reactivity, providing a general design principle for self-immolative triggers and conformational switches (Figure 4).

**Figure 4:**
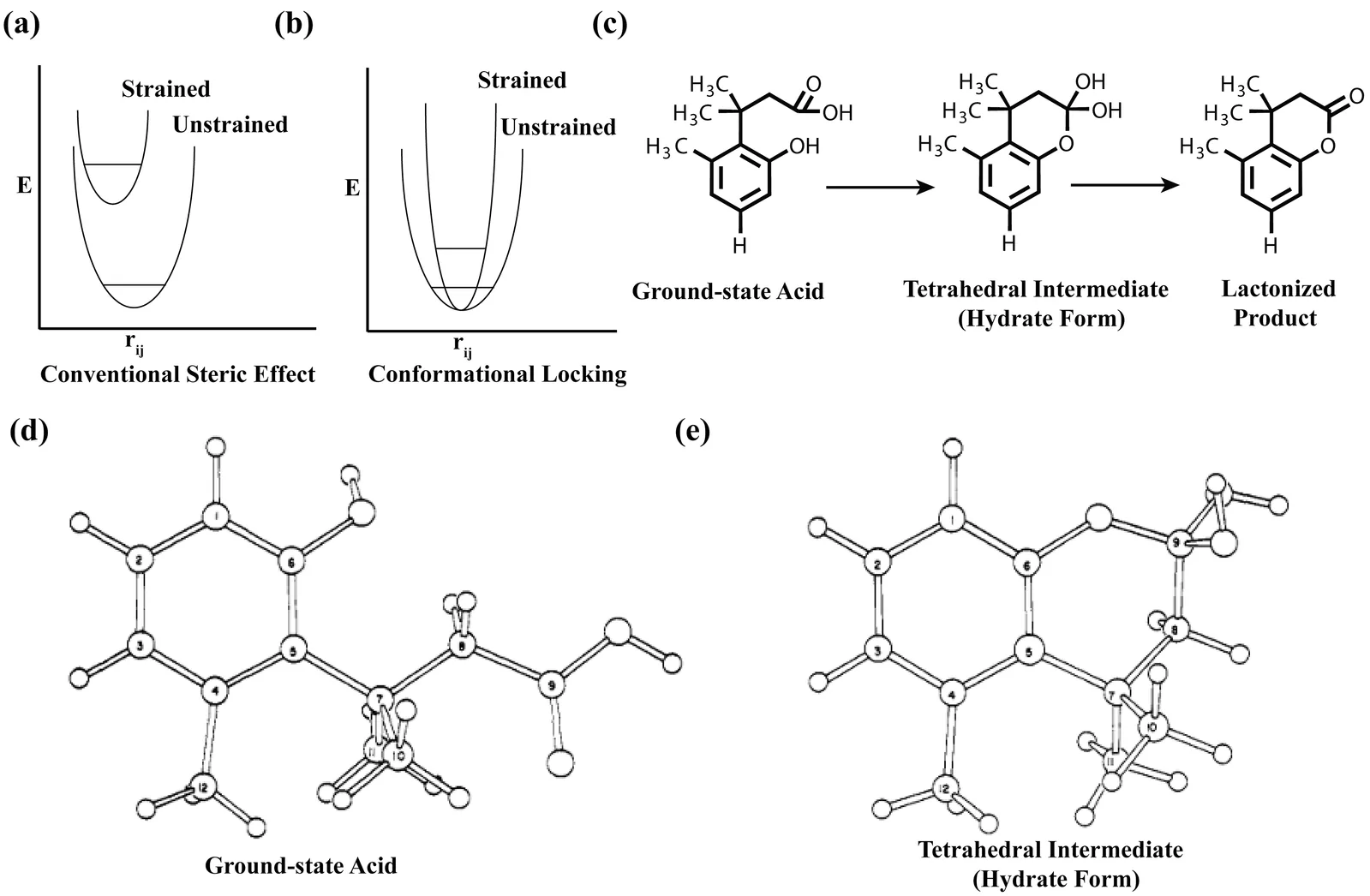
Comparison of steric strain relief and conformational locking models in hydroxy acid lactonization. (a) Conventional steric effect, where elevated ground-state strain is relieved upon lactonization. (b) Conformational locking, where preorganization reduces entropic penalties without significantly altering ground-state potential minimum. (c) Reaction pathway from ground-state acid through a tetrahedral intermediate to the lactonized product. (d, e) ORTEP representations of the calculated ground-state acid and tetrahedral intermediate, showing structural rearrangements consistent with steric strain relief. In (a, b), *E* denotes relative potential energy, *r* is a generalized conformational coordinate, and *i* and *j* are different conformational states. Panels (a, b) adapted from ref 36; (d, e) reproduced from ref 36. Copyright 1976 American Chemical Society.

Together, these mechanistic features, selective quinone reduction, rapid self-immolative release, and efficient signal generation, enable hNQO1-responsive probes to be systematically optimized. Early designs established feasibility, while subsequent generations addressed limitations in contrast, selectivity, spectral range, and translational performance.

## 4 First-Generation, hNQO1-Activatable Probe

The first step in translating this framework into a practical imaging platform was demonstration that intracellular hNQO1 activity is converted into a selective fluorescence turn-on response in living cells. Such feasibility established that triggered quinone, enzyme-activated reporter production may generate sufficient contrast (TBR) to differentiate hNQO1-positive and hNQO1-negative cells via intracellular hNQO1-initiated release of a strongly emissive naphthalimide (**NI**) reporter having diminished oxidative/reductive photoinduced electron transfer (OeT/ReT) quenching vs its probe counterpart, Figure 5.^1^ The **Q**_**3**_ quinone trigger is rapidly reduced by hNQO1 to a hydroquinone in the presence of NADH, demonstrating that enzyme-triggered activation can produce high-intensity fluorescence signals across cancer cell types. The effectiveness of this probe–reporter pair arises from its OeT/ReT quenching traits coupled with hNQO1-mediated activation, enabling high contrast between hNQO1-positive and -negative cells (Figure 5d,e). This design established a general framework for probes targeting intracellular cancer-associated enzymes, making it broadly applicable to tumors with elevated hNQO1 expression.

**Figure 5:**
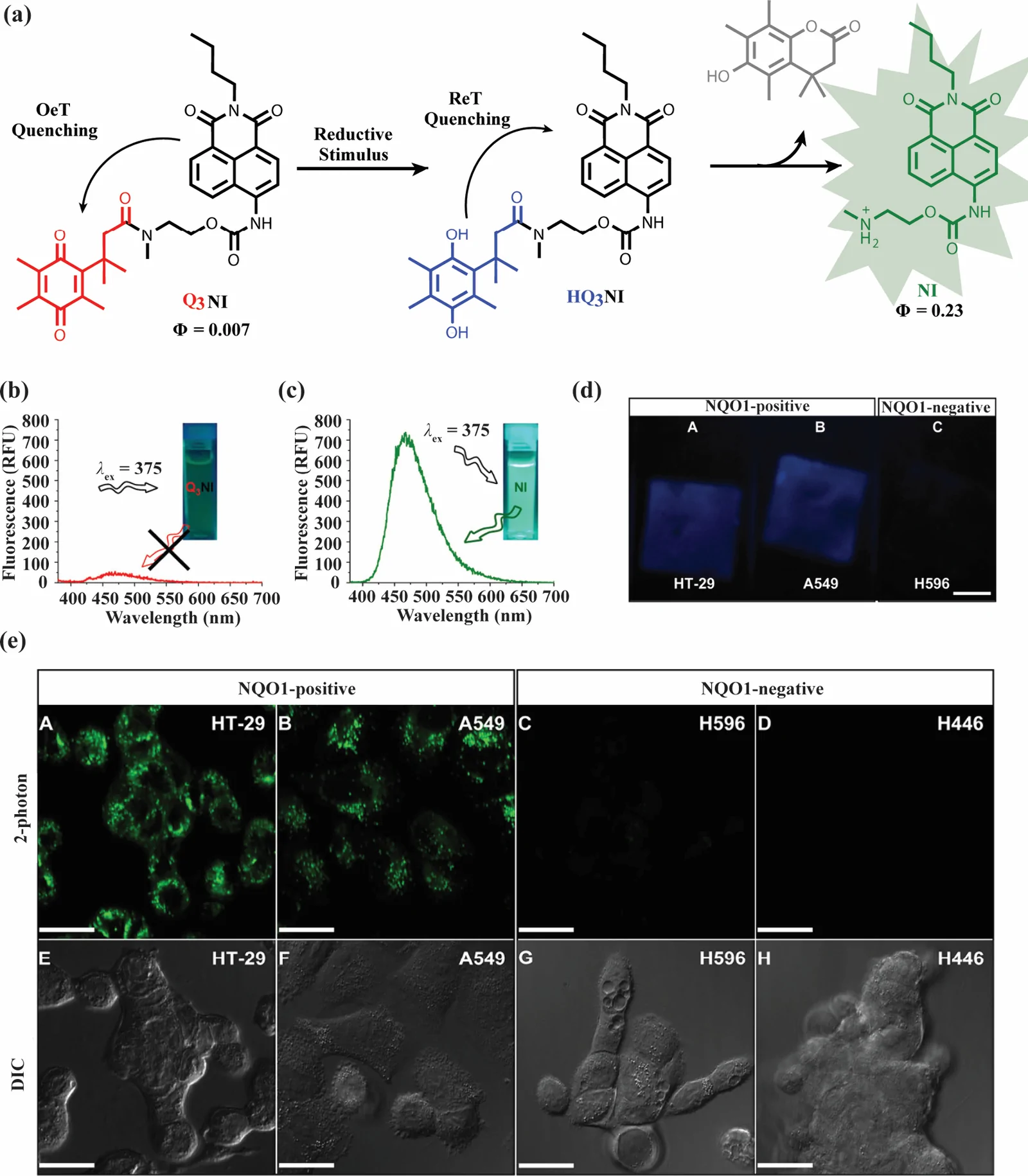
Traits, photophysical characterization, and cellular imaging of the hNQO1-activatable probe **Q**_**3**_ **NI**. (a) Quinone-mediated OeT quenching and activation via chemical or hNQO1 reduction to fluorescent **NI**. (b) and (c) Fluorescence spectra (λ_ex_ = 375 nm) and cuvette images (λ_ex_ = 365 nm) of 2.0 × 10^–6^ M **Q**_**3**_ **NI** and **NI** in pH 7.4, 0.1 M PBS. (d) Visual differentiation of hNQO1-positive HT-29 (A) and A549 (B) and hNQO1-negative H596 (C) *cell monolayers* after a 10 min incubation with 2.0 × 10^–5^ M **Q**_**3**_ **NI** (λ_ex_ = 365 nm); scale bar = 1 cm. (e) Two-photon confocal images of HT-29 (A and E), A549 (B and F), H596 (C and G), and H446 (D and H) cells incubated with 2.0 × 10^–5^ M **Q**_**3**_ **NI**; fluorescence images (top row) and corresponding differential interference contrast (DIC) images (bottom row) were acquired at λ_ex_ = 750 nm (3% laser power) without washing prior to imaging. Scale bar = 25 μm. Reproduced from ref 1. Copyright 2013 American Chemical Society.

Quantitatively, the use of the probe–reporter pair yields high TBRs with 9–23-fold differences in hNQO1-positive cell lines (A549, HT-29) and -negative lines (H596, H446), significantly exceeding the 2.5–5-fold contrast typical of earlier exogenous probes.^37^ Consequently, **Q**_**3**_ **NI** enables rapid detection and quantification of hNQO1-positive cells using flow cytometry, fluorescence microscopy, and two-photon imaging.^1^ Together, these results establish **Q**_**3**_ **NI** as a foundational platform for next-generation hNQO1-activatable imaging and theranostic systems.

Although **Q**_**3**_ **NI** use demonstrated differentiation of cell monolayers with/without hNQO1, a key limitation was revealed: similar probe and reporter spectra and energies make **Q**_**3**_ **NI** unsuitable for tissues and restrict image contrast. This motivated development of linker-engineered designs using self-immolative energy-shifting linkers, thereby improving off–on contrast and tissue mimic imaging.

## 5 Linker-Induced Alterations in the Spectrochemical Characteristics of Activatable Probes

As a first-generation hNQO1-probe, **Q**_**3**_ **NI** demonstrated that a photoinduced electron transfer (PeT)-quenched, quinone-masked probe could act as a visualization tool for hNQO1-overexpressing cells by selective hNQO1 activation to release the **NI** fluorophore that emits strong fluorescence. Even though this probe–reporter duo offers a transition from the “off” state to the “on” state upon **NI** reporter generation, the spectral traits of the intact probe and the released reporter are quite similar, leading to a modest TBR. To address this, a subsequent article^2^ reframes the main design strategy of hNQO1 probes through a tripartite architecture, leading to a wavelength-shiftable probe.^2^ This work moved the field toward attaining the ideal characteristics of a probe–reporter pair, namely, having a large separation in the absorption (λ_abs_^Probe^ vs λ_abs_^Reporter^) and emission (λ_em_^Probe^ vs λ_em_^Reporter^) maxima, so that the probe is essentially “invisible” in the excitation/emission regions of the reporter, thereby significantly increasing the TBR.^38,39^

Incorporating a tripartite probe design strategy, as evidenced by the **Q**_**3**_ **NI­(530)** probe, proved to be a highly successful strategy to make a wavelength-shiftable probe/reporter pair. The probe consists of the enzyme-triggerable **Q**_**3**_, a self-eliminating *N*-methyl-*p*-aminobenzyl alcohol (NMPABA) linker, and a near-silent masked naphthalimide fluorophore. Upon its selective and rapid hNQO1 activation and cyclizative removal (Figure 6a), autonomous elimination of the NMPABA linker yields the free unmasked naphthalimide reporter **NI­(530)** that has red-shifted spectral properties vs those of the **Q**_**3**_ **NI­(530)** probe (large Δλ_abs_ and Δλ_em_ for probe and reporter). The large difference in probe/reporter brightnesses (εΦ, Figure 6b,c) collectively translates into a significantly greater TBR contrast in hNQO1-positive vs hNQO1-negative cells, while also affording minimal background from scattered excitation light in imaging applications.

**Figure 6:**
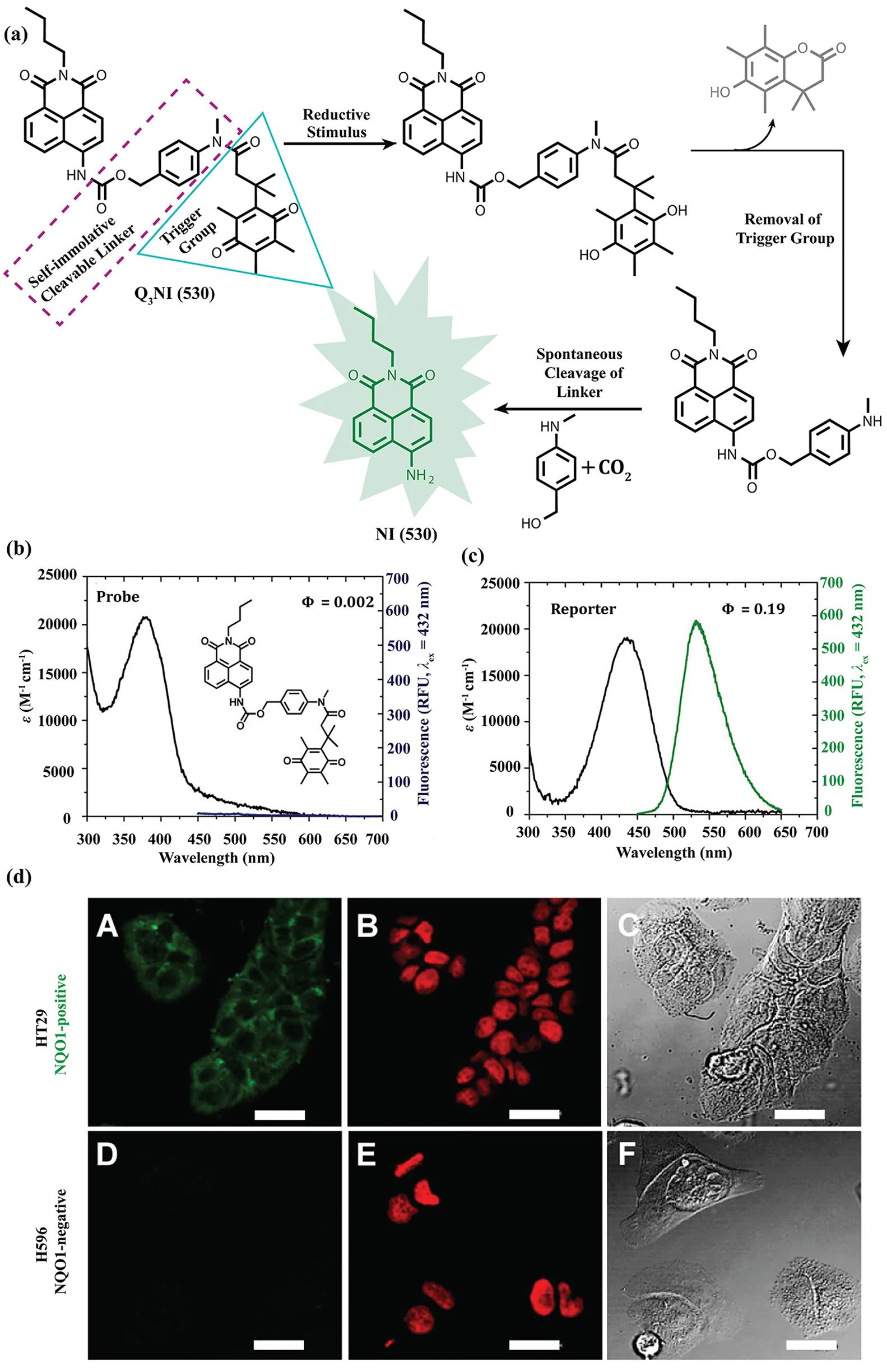
Mechanistic design, photophysical characterization, and cellular validation of the self-immolative, hNQO1-responsive **Q**_**3**_ **NI­(530)** probe. (a) Turn-on, self-immolative profluorophore (probe) and its enzyme-triggered reporter regeneration mechanism; hNQO1 reduction initiates fragmentation, releasing the fluorescent reporter. (b) and (c) Absorbance and emission spectra of 2 μM probe and reporter in pH 7.40, 0.1 M phosphate buffer at 25 °C. (d) Confocal images of hNQO1-positive HT-29 colorectal cancer cells (A–C) and hNQO1-negative H596 lung cancer cells (D–F) following incubation with 20 μM **Q**_**3**_ **NI (530)** at 37 °C for 30 min and 2 h, respectively. Probe fluorescence: A and D; DRAQ5 nuclear staining: B and E; differential interference contrast: C and F. Scale bar = 20 μm. Reproduced from ref 2. Copyright 2014 American Chemical Society.

Strikingly, the optimized probe design of **Q**_**3**_ **NI­(530)** led to observation of a large fluorescence turn-on in hNQO1-positive cells compared to hNQO1-negative cells (positive-to-negative ratio, aka TBR, ∼500:1). Importantly, the self-immolative linker-free **Q**_**3**_ **NI** probe afforded a TBR of only about 23. The 22-fold improvement in the TBR for the **Q**_**3**_ **NI­(530)** probe is attributed to the tripartite, linker-engineered design strategy, yielding a 95-fold fluorescence increase upon hNQO1 activation of the probe’s “off” state to yield the free reporter’s “on” state (Figure 6d). Furthering this is the 6-fold difference in extinction of the reporter and probe at the excitation maximum of the reporter (ε, Figure 6b,c). The significant separation in the absorption maxima of the **Q**_**3**_ **NI­(530)** probe and **NI­(530)** reporter leads to the near-silent probe absorbing less light in the excitation region of the reporter, hence minimizing the background signal (Figure 6b,c). The internal charge transfer (ICT) tunable scaffold of the **Q**_**3**_ **NI­(530)** probe and its analogues are currently being expanded to enable multiphoton imaging modalities and to incorporate alternative trigger groups targeting other disease-relevant enzymes and cellular dysfunction markers.^40^ Such efforts are directed toward the long-term objective of developing “intelligent” fluorescent probes for fluorescence-guided surgical resection and for evaluating the efficacy of personalized therapeutic strategies.^41,42^

The substantial linker engineering significantly improved contrast, demonstrating that the probe architecture can control both activation chemistry and imaging performance. Translating these gains to biological settings requires the evaluation of probe behavior in the presence of endogenous reductants and reductase enzymes to ensure that the fluorescence turn-on is selective for hNQO1.

## 6 Selectivity Characterization through Comparative Reductase Response

After improving the optical performance of hNQO1-activatable probes, the next critical issue was securing selectivity of these systems so that they are reliable reporters of hNQO1 activity in the face of cellular environments containing numerous endogenous reductants and quinone-processing enzymes that could potentially activate fluorogenic probes.

To set the gold standard for the maximum rate of probe activation and examine the impact of reduction potential of possible exogenous agents in the context of practical biological applications, probe activation by chemical species was assessed. Studies of the **Q**_**3**_ **MJSNR** probe with the strong reductant sodium dithionite (NaS_2_O_4_) showed quick and efficient reduction of the **Q**_**3**_ trigger to its hydroquinone form, followed by release of the fluorescent rhodamine reporter **MJSNR** (Figure 7a).^3^ In contrast, glutathione (GSH), ascorbic acid (AA), and dithiothreitol (DTT) did not activate the probe under the tested conditions (Figure 7b), while **Q**_**3**_ **MJSNR** reacted exceedingly slowly with NADH (*t*_1/2_ = 35.0 h; Figure S5 of the original report), supporting its resistance to rapid nonspecific reduction.

**Figure 7:**
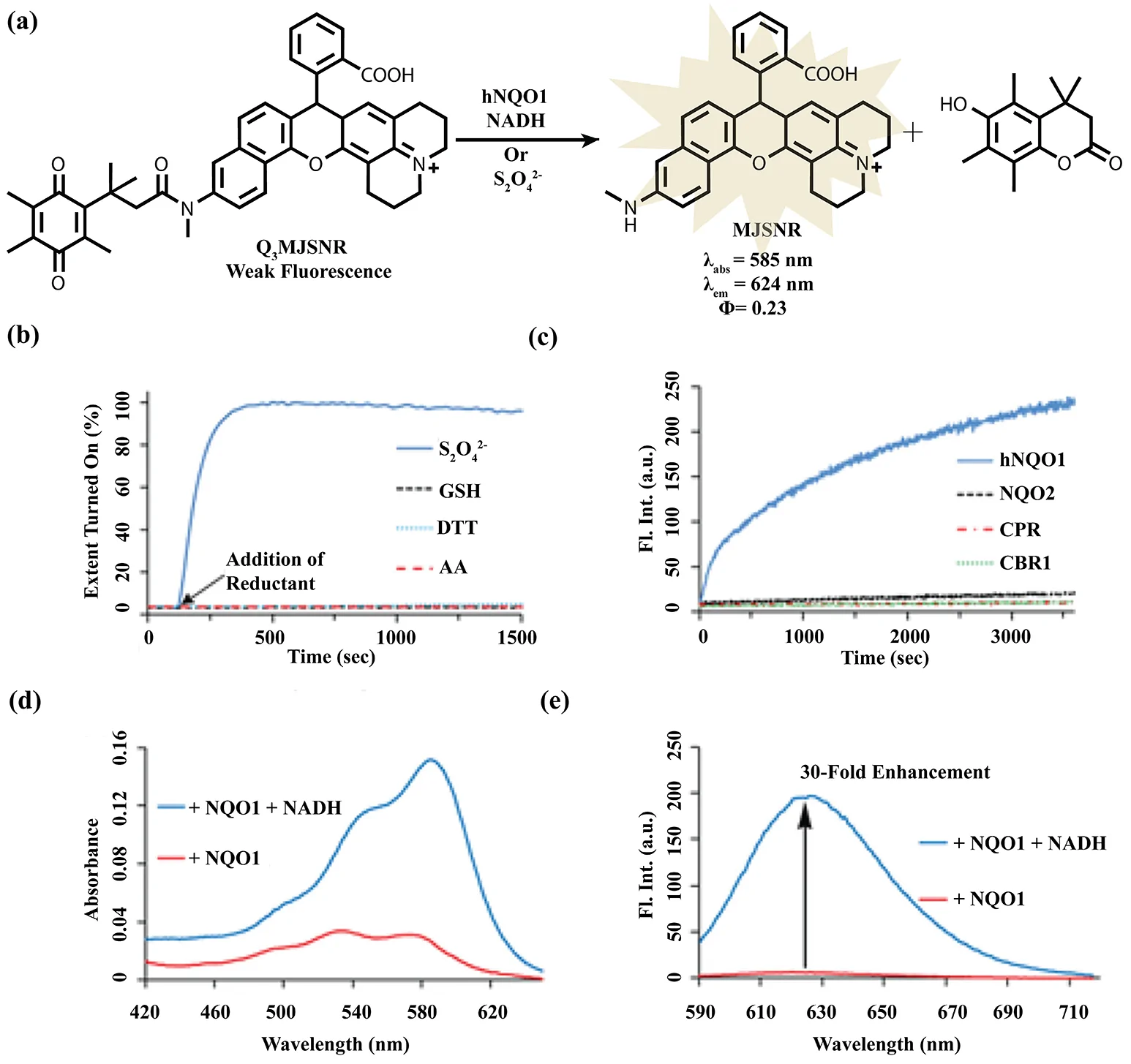
Mechanistic activation and enzymatic selectivity of the hNQO1-responsive probe **Q**_**3**_ **MJSNR**. (a) Reaction scheme illustrating release of the fluorescent reporter **MJSNR** following reduction of **Q**_**3**_ **MJSNR** by hNQO1 or chemical reductant (dithionite, S_2_O_4_^2–^). (b) Activated fluorescence of 5 μM probe by 1.6 mM dithionite with a negligible response to other common reductants (1 mM), demonstrating chemical selectivity. Probe stability toward NADH was evaluated separately (*t*_1/2_ = 35.0 h; Figure S5 of the original report).^3^ (c) Enzymatic activation of 5 μM probe by hNQO1 with NADH and probe response to NQO2 with its reduced synthetic cofactor (1-carbamoylmethyl-1,4-dihydronicotinamide) and CPR and CBR1 with NADPH, showing selective activation by hNQO1. (d) and (e) Activation of 5 μM probe by 0.75 μg (130 U) hNQO1 and 100 μM NADH in pH 7.4, 0.1 M PBS for 30 min. *T* = 25 °C. Adapted from ref 3. Copyright 2016 American Chemical Society.

Because cells contain multiple quinone-processing reductases, probe selectivity against competing enzymes was also evaluated under cofactor-supported assay conditions (hNQO1, 100 μM NADH; NQO2, 100 μM 1-carbamoylmethyl-1,4-dihydronicotinamide; CPR and CBR1, 100 μM NADPH). Enzymes capable of one- and two-electron quinone reduction, including carbonyl reductase-1 (CBR1),^43,44^ NRH:quinone oxidoreductase 2 (QR2, aka “NQO2”),^45^ and cytochrome P450 reductase (CPR)^46^ could in principle reduce the **Q**_**3**_ trigger and generate false-positive signals. However, the permethylated benzoquinone moiety of **Q**_**3**_ is effectively unreactive toward these reductases while remaining efficiently reduced by hNQO1 to release the fluorescent **MJSNR** reporter.

Although NQO2 produced a maximal signal increase of ∼9% (Figure 7c), the NQO2:hNQO1 activity ratio used in vitro (∼2.5)^3^ was ∼1,250-fold higher than the ∼0.002 ratio reported in human tumor cell lines.^47^ Similarly, the CPR:hNQO1 activity ratio used in vitro (∼2.9)^3^ was ∼73-fold higher than the ∼0.04 ratio reported in HT-29 tumor xenografts.^48^ These results support the high selectivity of **Q**_**3**_ **MJSNR** activation for hNQO1 (Figure 7c).

Further evidence for selective activation was obtained using dicoumarol, a known hNQO1 inhibitor.^49^ HT-29 colorectal cancer cells preincubated with dicoumarol showed markedly reduced fluorescence following **Q**_**3**_ **MJSNR** treatment (Figure 8). In separate experiments, pretreatment with quercetin, an NQO2 inhibitor, or rutin, a CBR1 inhibitor, produced little change in fluorescence (Figure S7 of the original report).^3^ Together, these studies demonstrate that **Q**_**3**_ probes retain high selectivity for hNQO1 even in chemically and biologically reducing environments, supporting their use as reliable imaging agents in complex cellular systems.^3^

**Figure 8:**
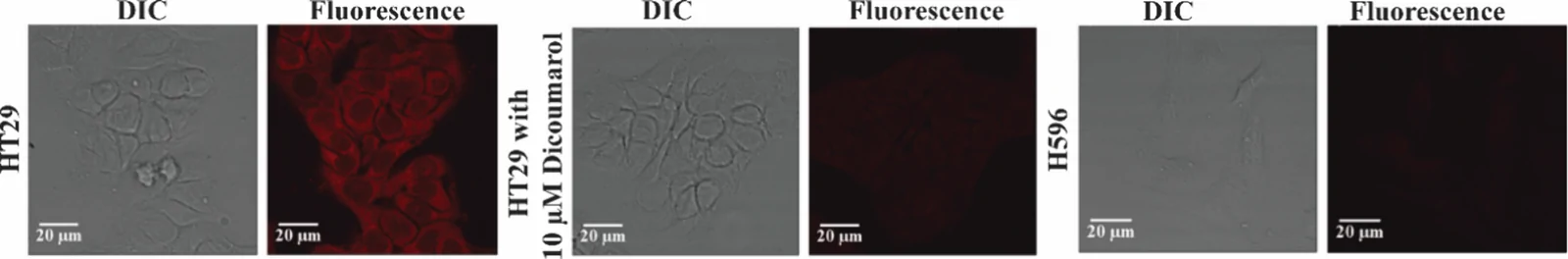
Confocal microscopy images of HT29 cells (hNQO1 positive, left panel) and H596 cells (hNQO1 negative, right panel) incubated with 5 μM **Q**_**3**_ **MJSNR** for 10 min at 37 °C; HT29 cells (hNQO1 positive, center panel) pretreated with 10 μM dicoumarol inhibitor of hNQO1. Fluorescence images are on the right side and differential interference contrast (DIC) images on left of each panel. Cells were excited with a 543-nm HeNe laser (laser intensity = 48%). Scale bar = 20 μm. Reproduced from ref 3. Copyright 2016 American Chemical Society.

With high selectivity established, the next challenge is extending performance to more demanding imaging settings, where tissue penetration, autofluorescence, and optical scattering limit the performance.

## 7 Enhancing Imaging Performance with Wavelength-Shiftable NIR Probes

Once hNQO1-responsive probes demonstrated intracellular activation and enzymatic selectivity, the next limitation was the optical constraints of UV–visible fluorophores. Although effective in cultured cells, these shorter-wavelength systems are less suitable for tissue imaging because of strong scattering, shallow penetration, autofluorescence, phototoxicity, and photobleaching.^50^ These limitations motivated the development of wavelength-engineered probes operating in the near-infrared (NIR) region.

Most hNQO1-activatable probes employ reporters emitting in the UV–visible range (250–650 nm), where biological tissues strongly absorb and endogenous biomolecules exhibit substantial autofluorescence.^51^ In contrast, the NIR region (∼650–900 nm) enables deeper penetration, reduced scattering, and lower background fluorescence because tissue components absorb less light in this spectral window (Figure 9).^52,53^

**Figure 9:**
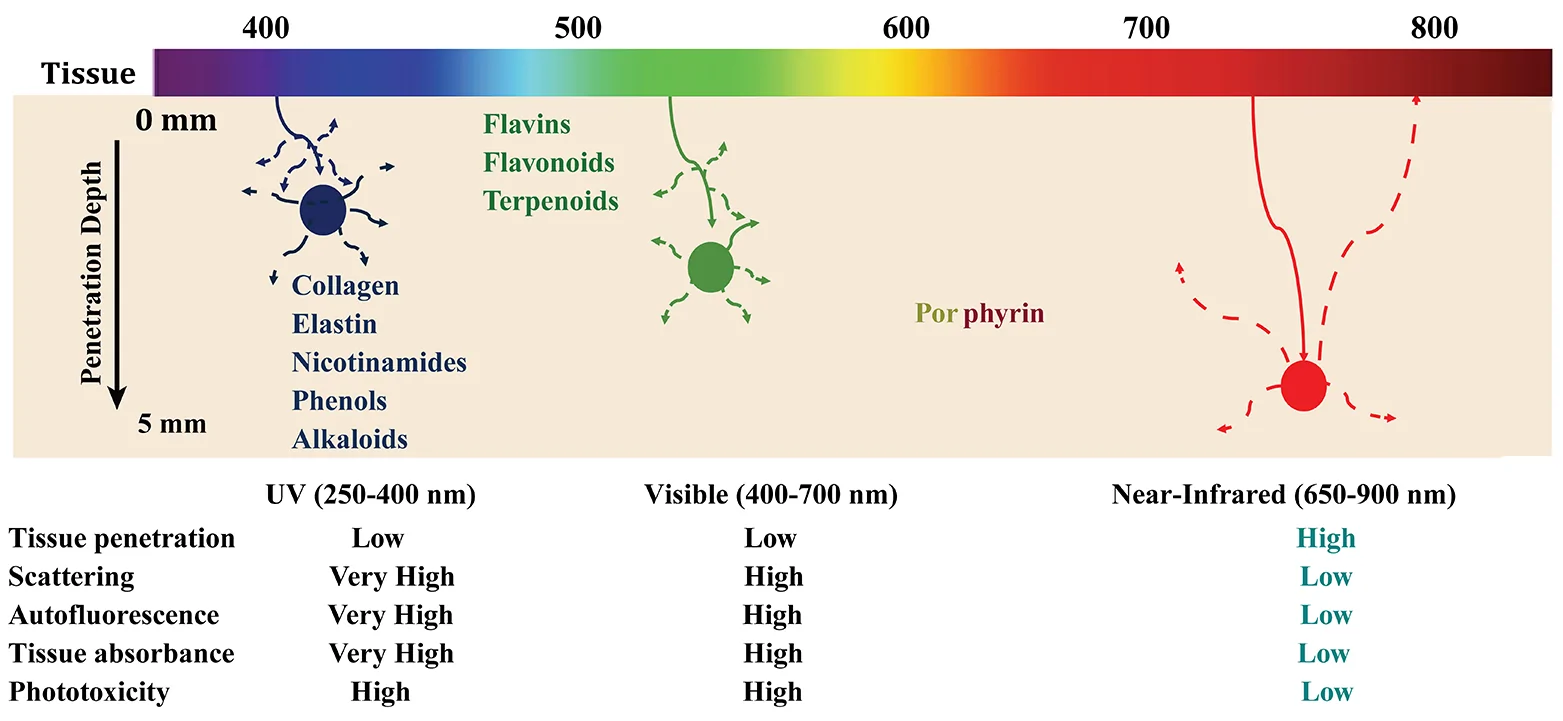
Comparison of optical properties across UV (250–400 nm), visible (400–650 nm), and near-infrared (650–900 nm) regions. Adapted with permission from ref 53. Copyright 2022 Elsevier.

The **Q**_**3**_ **STCy** probe (λ_abs_^Q_3_STCy^ = 786 nm, λ_em_^Q_3_STCy^ = 798 nm) exploits this NIR strategy using a cyanine-based reporter (TCy). The intact probe is strongly PeT-quenched and essentially nonemissive, whereas enzymatic activation releases the fluorescent **TCy** reporter (λ_abs_^TCy^ = 606 nm, λ_em_^TCy^ = 755 nm).^20^ Additionally, the **TCy** reporter has a remarkable Stokes shift (∼149 nm) due to the ICT effect. Therefore, **Q**_**3**_ **STCy**/**TCy** is also a wavelength-shiftable NIR system (Figure 10a). Upon hNQO1-catalyzed activation, **Q**_**3**_ **STCy** offers several-fold fluorescence enhancement (Figure 10b,c) and high TBR (∼3–5), enabling a clear distinction between hNQO1-positive and deficient cancer cells by optical microscopy (Figure 10d).

**Figure 10:**
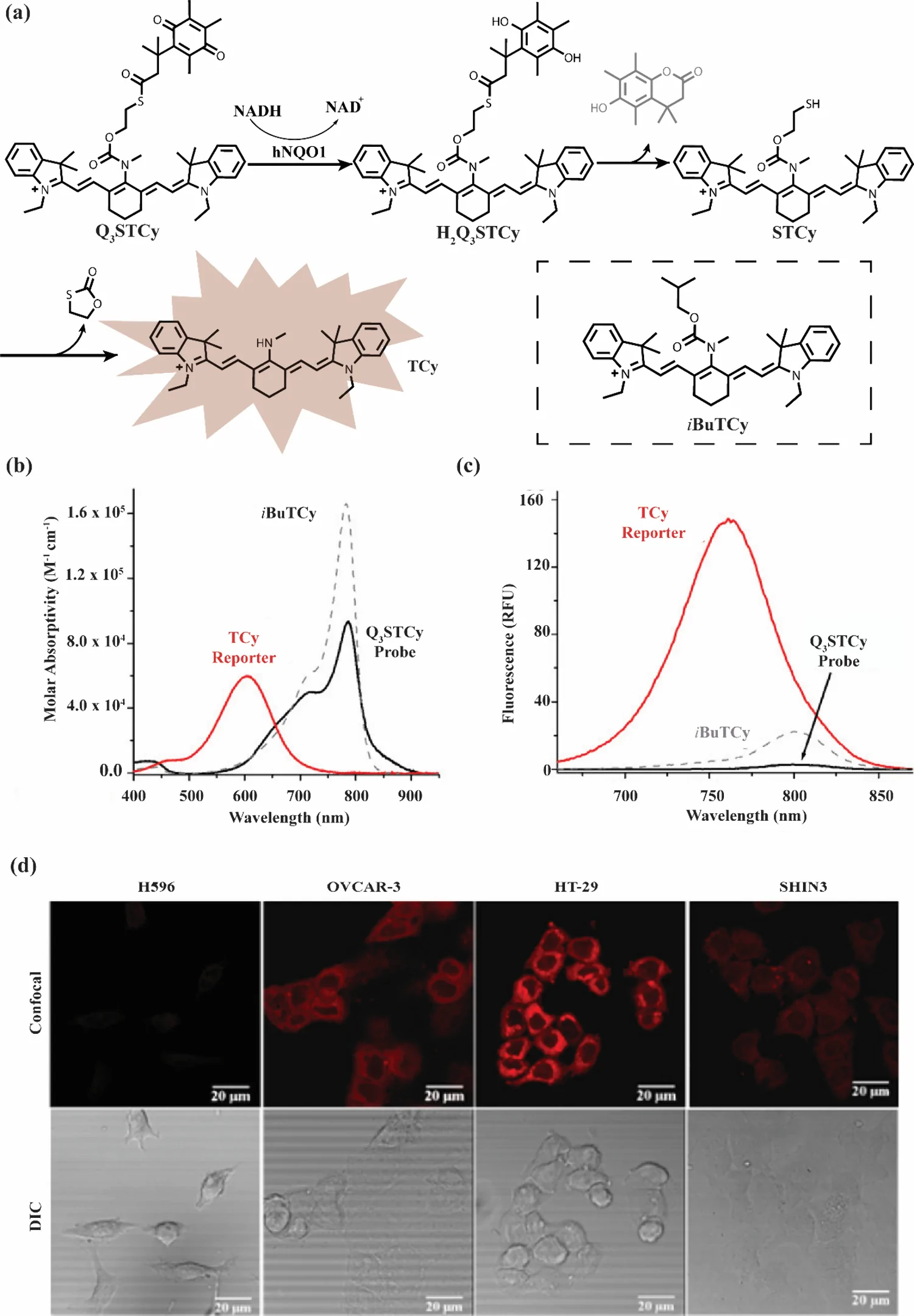
Mechanistic design, photophysical characterization, and cellular demonstration of the hNQO1-responsive NIR probe **Q**_**3**_ **STCy**. (a) Enzyme-triggered release of the **TCy** reporter from **Q**_**3**_ **STCy** following reduction by hNQO1, resulting in fluorescence turn on. (b and c) Absorption and emission spectra of **TCy**, **Q**_**3**_ **STCy**, and *i* **BuTCy** (2 μM in 1% DMSO/0.1 M PBS/0.1 M KCl, pH 7.4, 25 °C), demonstrating the spectral difference between probe and reporter (λ_ex_ = 600 nm for emission). (d) Confocal fluorescence and corresponding DIC images of hNQO1-negative H596 and hNQO1-positive OVCAR-3, HT-29, and SHIN3 cells following incubation with 5 μM **Q**_**3**_ **STCy** (30 min, 37 °C); selective fluorescence enhancement is observed in enzyme-positive cells. Scale bar = 20 μm. Reproduced from ref 20. Copyright 2017 American Chemical Society.

A major advance was demonstrating probe utility in three-dimensional (3D) multicellular tumor spheroids (MCTSs), which better mimic solid tumor architecture.^54–57^ In colorectal MCTSs, **Q**_**3**_ **STCy** revealed spatially heterogeneous hNQO1 activity with strong NIR fluorescence at the periphery and weaker signals in the hypoxic core of 970 mm HT-29 MCTSs (Figure 11).^20^ This capability is clinically relevant for detecting residual micrometastases following tumor resection.^58^ In a peritoneal ovarian carcinoma mouse model (POCM, SHIN3-DsRed), intraperitoneal administration of **Q**_**3**_ **STCy** selectively visualized submillimeter tumor nodules (Figure 12), demonstrating potential utility in fluorescence-guided surgery (FGS).

**Figure 11:**
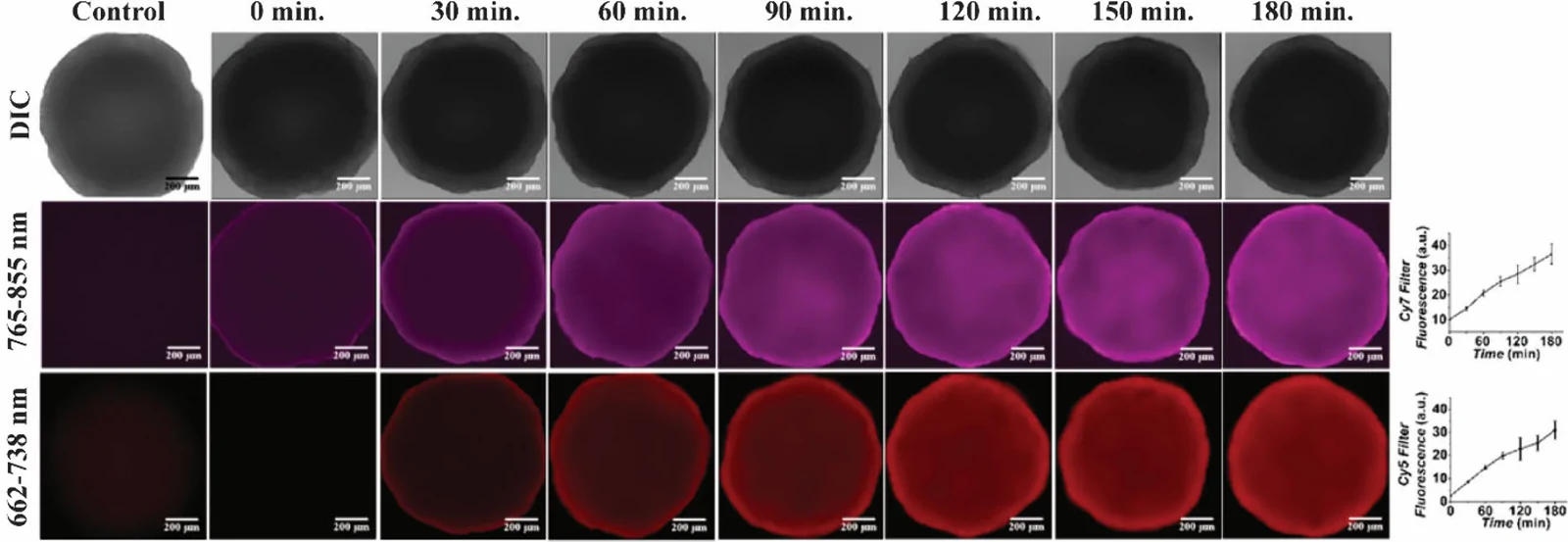
Time-dependent activation of **Q**_**3**_ **STCy** in *living* HT-29 multicellular tumor spheroids (MCTSs). Wide-field fluorescence and DIC micrographs of 23-day-old spheroids (970 ± 24 μm diameter) following incubation with 5 μM **Q**_**3**_ **STCy** in RPMI-1640 medium at 37 °C; scale bar = 200 μm. Images were acquired at 30 min intervals using Cy7 and Cy5 filter sets. Progressive fluorescence enhancement is observed over time, while DIC images reveal the characteristic necrotic core (dark central region). The Control corresponds to spheroids maintained in probe-free medium. Quantified fluorescence intensities represent the mean of four spheroids (±1 SD). Reproduced from ref 20. Copyright 2017 American Chemical Society.

**Figure 12:**
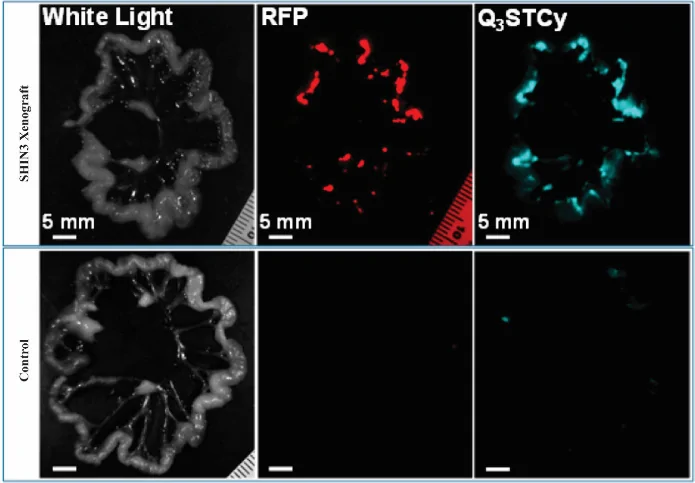
Fluorescence visualization of peritoneal metastases of SHIN3 human ovarian cancer cells (mouse model) following intraperitoneal administration of **Q**_**3**_ **STCy**. Spectral imaging of mesenteric tissue at 1 h postinjection (300 μL of 100 μM probe in pH 7.4 PBS) reveals probe-associated fluorescence localized to tumor regions. Red fluorescent protein (RFP) emission arises from SHIN3-DsRed-transfected cancer cells. Control animals without xenografts exhibit minimal probe-associated signals. Scale bar = 5 mm. Reproduced with permission from ref 20. Copyright 2017 American Chemical Society.

Extending hNQO1-responsive probes into the NIR region and validating them in spheroid and animal models advance the platform from proof-of-concept to clinically relevant imaging. This raises a key question: Can these probes compete with or complement existing imaging agents in diagnostic and surgical workflows?

## 8 Translational Potential of hNQO1-Responsive Probes for Biomedical Imaging

Strong performance in NIR cellular and animal imaging models represented a significant advance,^20^ but translational relevance requires more than a proof-of-principle signal generation. The key question is whether hNQO1-responsive probes can deliver practical diagnostic value and support clinically meaningful decisions versus those of established imaging approaches.

**Q**_**3**_ **STCy** was compared with the known green fluorescent probe gGlu-HMRG (*extracellular* membrane enzyme) using cells (Figure 13),^59^ ex vivo tissues, and mice. In SHIN3 ovarian cancer cells, both probes exhibited weak initial signals followed by increased activation after 1 h; however, **Q**_**3**_ **STCy** consistently showed higher fluorescence, *reflecting more effective intracellular activation*. In the peritoneal ovarian cancer metastasis mouse model (POCM; SHIN3-DsRed), fluorescence from both probes colocalized with DsRed tumor nodules; controls showed no co-occurrence. **Q**_**3**_ **STCy** detected small metastases with high sensitivity (∼97%) but moderate specificity (∼61%); the latter attributed to background signals from intestinal and intra-abdominal tissues (Figure 14).^4^

**Figure 13:**
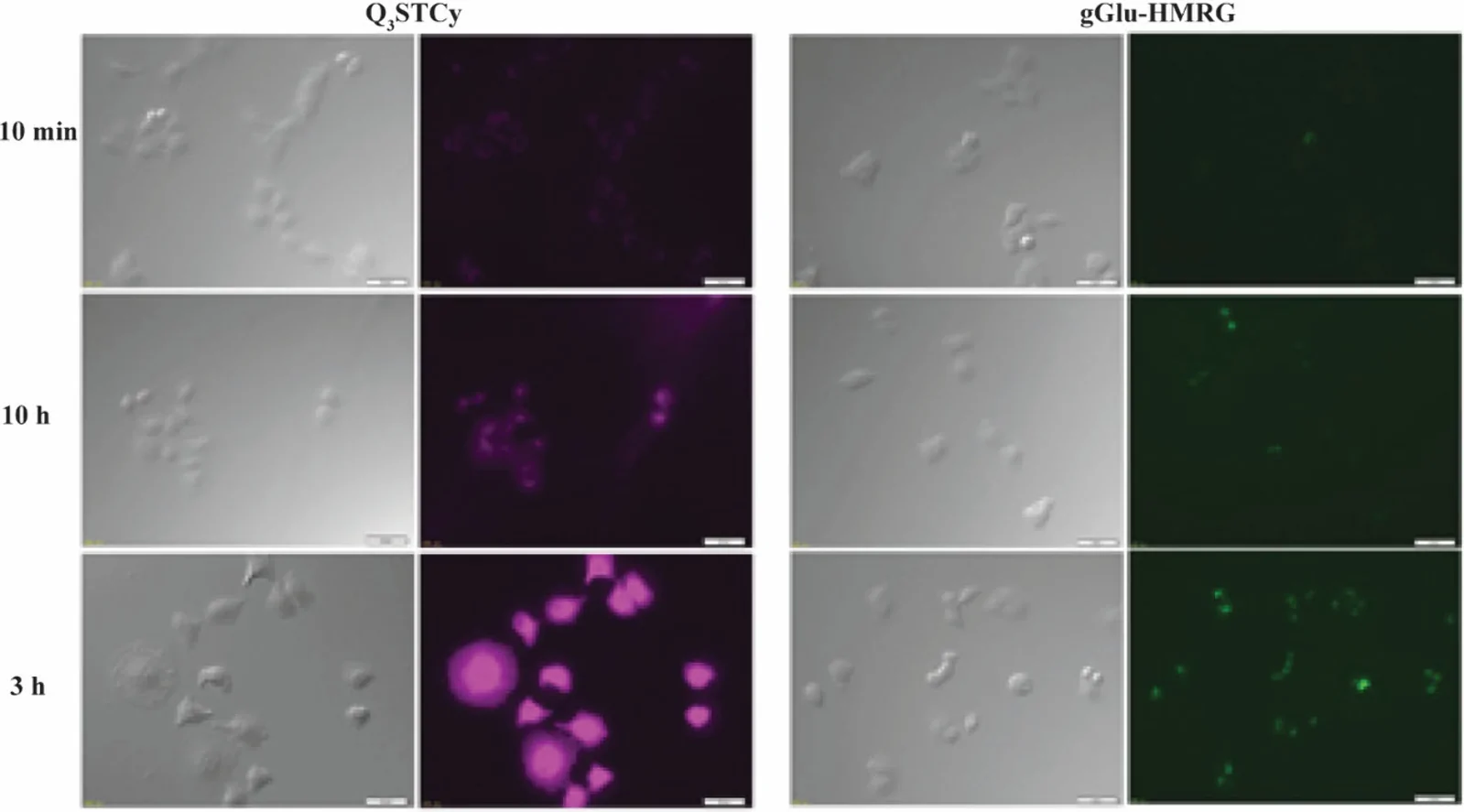
Fluorescence microscopy images of SHIN3 human ovarian cancer cells after incubation with 2 μM **Q**_**3**_ **STCy** or **gGlu-HMRG** for 3 h. Under the respective imaging conditions, **Q**_**3**_ **STCy**-treated cells exhibited higher cellular fluorescence than **gGlu-HMRG**-treated cells. Scale bar = 50 μm. Reproduced with permission from ref 4. Copyright 2017 Rapamycin Press.

**Figure 14:**
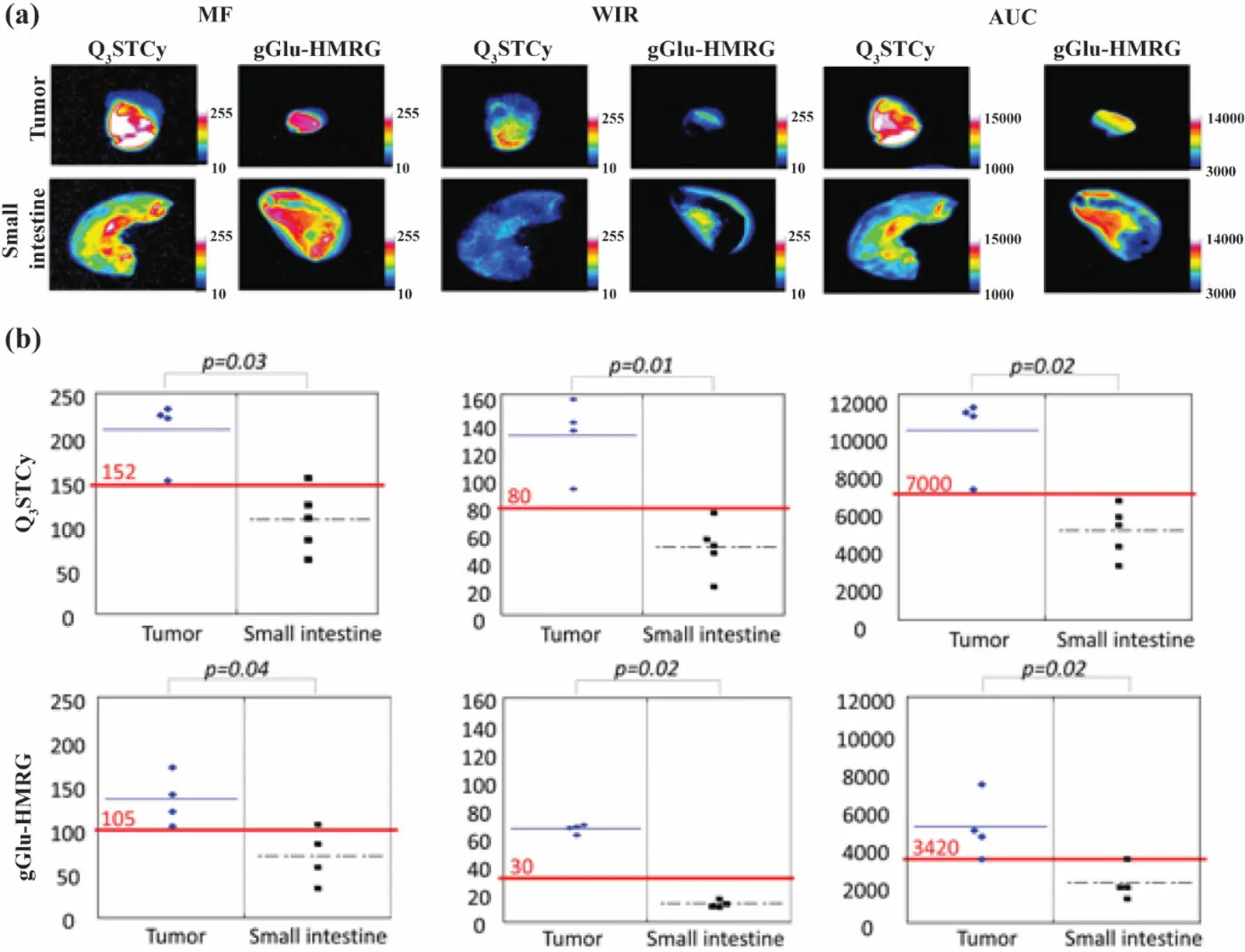
Kinetic analysis of activatable fluorescence probe emission for differentiating tumor and normal tissue. (a) Kinetic maps derived from dynamic fluorescence imaging, including maximum fluorescence (MF), wash-in rate (WIR), and area under the curve (AUC), show both **Q**_**3**_ **STCy** and **gGlu-HMRG** produce signal intensities in tumor regions vs healthy small intestine that offer clear visual discrimination. (b) Quantitative analysis of kinetic parameters (MF, WIR, and AUC) for each probe. Red lines and corresponding values indicate the threshold criteria used for tumor identification. Reproduced with permission from ref 4. Copyright 2017 Rapamycin Press.

Ex vivo imaging of extracted living tissues revealed rapid signal increases in tumors for both probes, whereas **Q**_**3**_ **STCy** showed a slower increase in bowel compared to minimal changes for gGlu-HMRG. Importantly, the use of kinetic imaging approaches (e.g., maximum fluorescence (MF), wash-in-rate (WIR), and area under curve (AUC)) led to significantly higher tumor signals for both probes.^60^ The use of **Q**_**3**_ **STCy** with WIR and AUC approaches yielded 100% sensitivity and 100% specificity for the micrometastases. For gGlu-HMRG, these values were also 100% and 100%. *It is very important to note that* ***Q***_***3***_ ***STCy*** *produces diagnostically meaningful contrast within 10 min of topical administration, which lasts for at least 60 min*, in comparison to the signal being 1.2–1.8 times lower in the entire 60 min duration and nonsignificant at 60 min for the extant extracellularly activated gGlu-HMRG probe. Thus, integrating kinetic mapping with the **Q**_**3**_ **STCy** probe traits offers high-sensitivity (100%) and high-specificity (100%) detection of tiny tumors.^4^ Additionally, nonspecific bowel signals were attributed to serum-induced background noise, anticipated to be modest at clinically relevant lower doses of **Q**_**3**_-based probes.

These results demonstrate that combining hNQO1-activatable NIR probes such as **Q**_**3**_ **STCy** with kinetic mapping enables sensitive and specific tumor imaging, overcoming limitations of green-emitting probes like gGlu-HMRG, which include shallow penetration and autofluorescence.^4^ The hNQO1-based NIR probe approach supports detection of submillimeter (≤2 mm) metastases and provides a practical route toward fluorescence-guided surgery.

## 9 Challenges in hNQO1-Activatable Probe Design and Applications

As hNQO1-responsive probes advance toward translation, limitations shift from feasibility to practical factors governing robustness, reproducibility, and clinical comparability.^12,61^ Progress, therefore, depends on addressing challenges in stability, specificity, delivery, and standardization (Figure 15). Despite broad interest, unresolved issues limit deployment in complex biological systems.^1,3,20,62–65^ Chemical robustness remains challenging: trigger scaffolds must remain stable in biological media yet activate rapidly in the cytosol, while avoiding hydrolysis, nonspecific interactions, protein binding, and aggregation-induced quenching.^3,65–67^ Bioavailability and delivery further complicate application, as performance depends on controlling lipophilicity, aggregation, serum stability, and pharmacokinetics across topical, oral, and intravenous routes. False activation remains a challenge, as Nrf2-responsive hNQO1 can be upregulated in nonmalignant conditions (e.g., oxidative stress, inflammation), potentially leading to false positives. Structure–activity relationships are also difficult to generalize, as small structural changes, comedications, and enzyme inhibitors can alter activation kinetics.^3,68^

**Figure 15:**
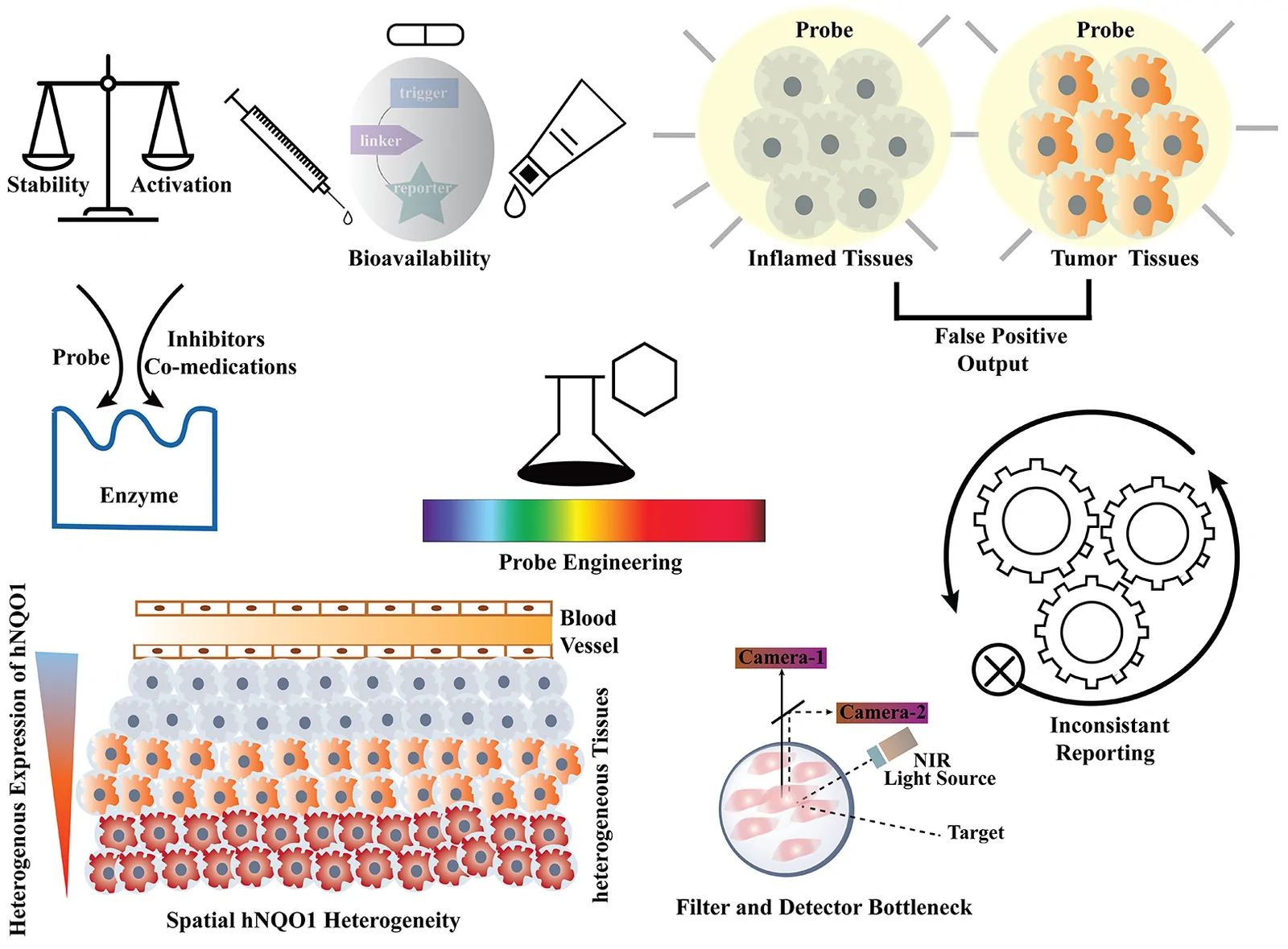
Unmet challenges in hNQO1-based imaging and activity quantification research.

Photophysical constraints remain to be significant. NIR-I/NIR-II emission improves penetration and reduces autofluorescence but often leads to lower quantum yield and increased nonradiative decay, aggregation, and photobleaching.^4,69^ Biological heterogeneity further complicates translation, as hNQO1 expression varies across tissues and within tumors.^70^ Genetic heterogeneity also affects translation: the common NQO1*2 polymorphism (rs1800566; p.Pro187Ser), particularly prevalent in Asian populations, causes near-absent NQO1 activity in homozygotes and reduced activity in heterozygotes; H596 cells are homozygous for this variant.^71,72^ Thus, clinical use may require genotype- and/or activity-based patient stratification. Instrumentation adds limitations: signal sensitivity decreases with depth, and although two-photon microscopy improves penetration, it requires ultrafast lasers that are not always compatible with clinical workflows; standardized detection and calibration for far-red/NIR-II imaging also remain uneven.^69^

## 10 Future Perspectives

Because hNQO1 activity increases in nonmalignant tissues, specificity in complex redox environments requires multiresponsive or logic-gated probes combining hNQO1 with additional biomarkers to enhance spatial selectivity while maintaining sensitivity.^73–76^ Ratiometric or orthogonal channels support quantitation and discrimination between tumor margins and other pathological states.

Although red-shifted emitters reduce autofluorescence and improve penetration,^77,78^ aggregation-induced quenching and reduced quantum efficiency^4,79^ require coordinated optimization with detector technologies, optical filters, and calibration. Alternative deployment strategies, including topical or transmucosal delivery, can circumvent systemic challenges,^4^ while quantitative fluorescence-guided surgery with defined target-to-background thresholds may accelerate clinical adoption.^80,81^ Biological validation must evolve; organoids, ex vivo tissues, and patient-derived spheroids improve translational relevance and reduce species transfer limitations,^82,83^ while inhibitor controls remain essential for confirming mechanism-specific activation.^84^

Beyond imaging, hNQO1-responsive chemistry supports theranostic integration via probe/antibody–drug conjugates, self-immolative prodrugs, and enzyme-responsive delivery systems, enabling sensing with controlled release.^34,85–89^

Minimum reporting guidelines, including structures, photophysical parameters and emission wavelengths, fluorescence lifetimes, activation kinetics, physicochemical and uptake characteristics, together with shared data sets and metadata, will enable fair benchmarking and support AI-assisted probe design.

## 11 Summary

This Account describes the development of hNQO1-activatable fluorogenic probes and design principles underlying their performance. Integration of quinone triggers, self-immolative linkers, and spectrally distinct reporters enables the transduction of enzyme activity into reliable optical signals in biological environments. Successive probe generations improved fluorescence contrast, spectral properties, and biological performance, demonstrating how design governs imaging capability and that activity-based probes reveal biochemical processes inaccessible through expression measurements. Continued integration with advanced imaging and translational validation may position hNQO1-responsive systems as platforms for mapping enzyme activity and guiding disease diagnosis and therapy.
